# Dysbiosis of intestinal microbiota in patients with neuromyelitis optica spectrum disorders

**DOI:** 10.3389/fimmu.2026.1747643

**Published:** 2026-02-27

**Authors:** Qin Du, Xiaofei Wang, Ziyan Shi, Hongxi Chen, Ying Zhang, Rui Wang, Zichao Mou, Lingyao Kong, Hongyu Zhou

**Affiliations:** Department of Neurology, West China Hospital, Sichuan University, Chengdu, Sichuan, China

**Keywords:** dysbiosis, gut microbiota, immunosuppressant, metabolomic profiles, neuromyelitis optica spectrum disorders

## Abstract

**Objective:**

This study aimed to explore the specific microbial signatures and metabolomic profiles of fecal microbiota in patients with neuromyelitis optica spectrum disorders (NMOSD) and assess the effects of immunosuppressants on their gut microbiota using a longitudinal cohort study.

**Methods:**

We enrolled 21 treatment-naïve NMOSD patients and 21 matched healthy controls (HCs). Fecal microbial composition and metabolomic profiles were compared between groups using 16S rRNA gene sequencing and ultra-high-performance liquid chromatography-mass spectrometry. Subsequently, fecal samples from NMOSD patients were collected and reassessed after immunosuppressant treatment.

**Results:**

The gut microbial composition and metabolomic profiles of NMOSD patients were distinct from those of HCs. The α-diversity metrics were significantly higher in NMOSD patients than in HCs (P <0.001). Microbiome alterations in NMOSD patients were characterized by increased abundances of *Streptococcus* and *Ruminococcus*, and decreased abundances of *Faecalibacterium*, *Ralstonia*, and *Pseudomonas* at the genus level (all with linear discriminant analysis scores > 4 and P < 0.001). Additionally, Phylogenetic Investigation of Communities by Reconstruction of Unobserved States analysis identified 19 differentially abundant metabolites and 44 altered metabolic pathways in NMOSD patients compared to HCs. Immunosuppressive treatment for over six months may reduce these differences, shifting the gut microbiota composition and metabolite profiles of NMOSD patients closer to those of HCs.

**Interpretation:**

Our study revealed significant gut microbiome dysbiosis and metabolic abnormalities in patients with NMOSD, which were markedly alleviated after six months of immunosuppressive treatment. These preliminary findings suggest the gut microbiota biomarkers could serve as potential therapeutic targets in the future.

## Introduction

Neuromyelitis optica spectrum disorder (NMOSD) is a severe inflammatory demyelinating disease of the central nervous system, characterized by recurrent attacks of transverse myelitis and optic neuritis (1). Although the etiology of NMOSD remains elusive, aquaporin-4 (AQP4) has been identified as a disease-specific serum autoantibody marker (2). Growing evidence indicates that environmental factors play a significant role in the pathogenesis and progression of inflammatory and autoimmune diseases.

The gut microbiota, often called the second brain, may influence brain activity through the gut-microbiota-brain axis under both physiological and pathological conditions. The gut microbiome contributes substantially to these disorders by affecting the immune system and metabolic pathways (3). Dysbiosis of gastrointestinal microbiota affects the differentiation of proinflammatory T-cells and may enhance organ-specific autoimmunity (4). Extensive homology between gut microbiota and AQP4 protein demonstrates molecular mimicry in the pathogenesis of NMOSD (5).Compared with healthy controls (HCs), antibody responses against *Helicobacter pylori* and gastrointestinal antigens were more frequently observed in NMOSD patients, suggesting that alterations in the gastrointestinal environment may contribute to the initiation or progression of NMOSD (6).Our previous research found that NMOSD patients had increased abundances of the pathogenic genera *Streptococcus* and *Flavonifractor* compared with HCs (7).

Previous studies have primarily examined group differences at a cross-sectional level, which may not fully account for treatment-related effects. To address this limitation, we conducted a case-control study comparing gut microbiome and metabolomic profiles between treatment-naïve NMOSD patients and HCs. Additionally, we performed longitudinal sampling (before and six months after immunosuppressant therapy) to evaluate how these medications alter the gut microbiota in NMOSD patients.

## Methods

### Study design and participants

**Figure 1:**
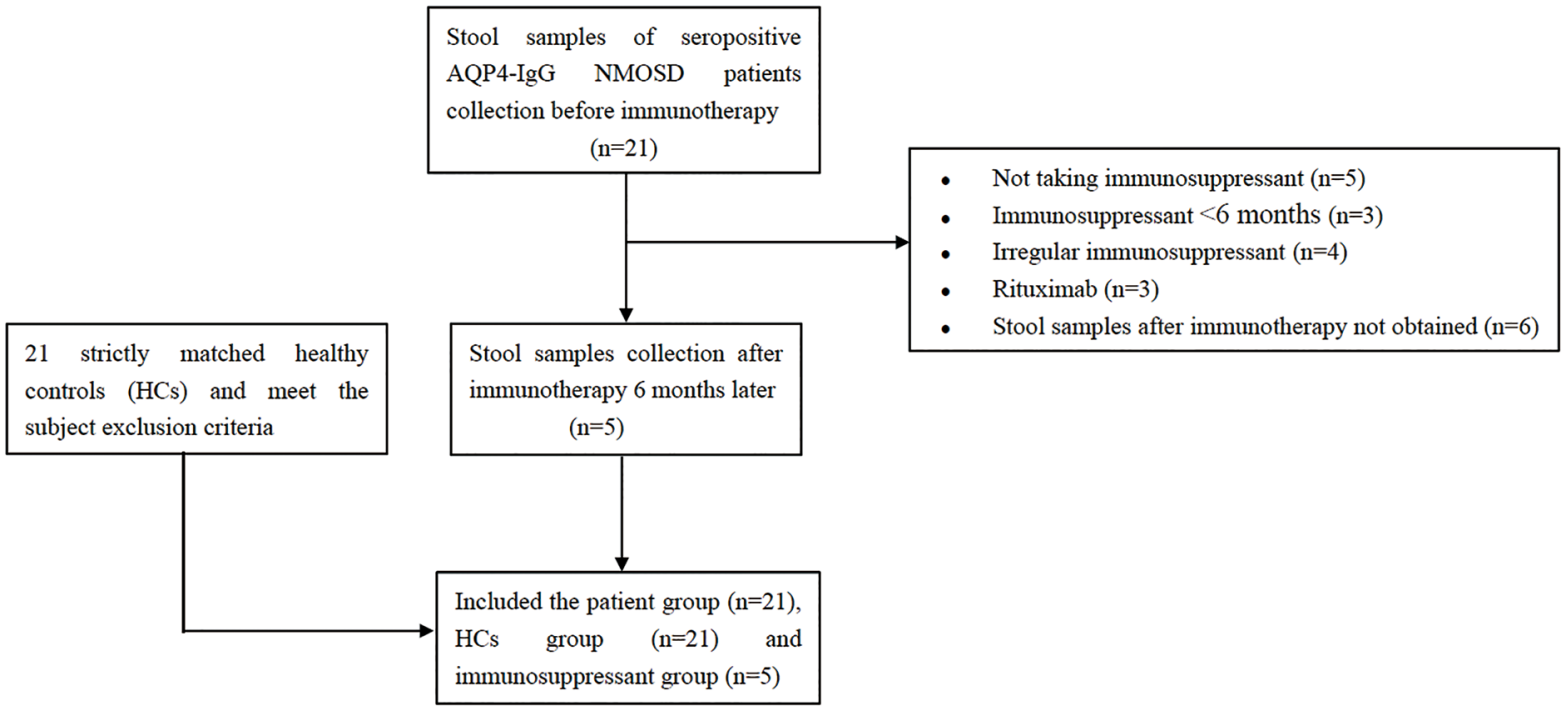
Flowchart of participant enrollment. The study included patients with NMOSD and HCs. NMOSD patients underwent assessments both before and after immunosuppressive therapy. HC, healthy control; NMOSD, neuromyelitis optica spectrum disorder.

A total of 42 subjects, comprising 21 treatment-naïve NMOSD patients (no treatment, NT group) and 21 HCs, were consecutively recruited in our study from West China Hospital, Sichuan University between January 2021 and June 2023. Patients were defined as treatment-naïve under two criteria: 1) being immunotherapy-naïve at initial diagnosis; or 2) having discontinued all immunomodulatory therapies after a continuous use of less than six months and maintaining a treatment-free washout period for at least six months. All patients met the 2015 international diagnostic criteria for NMOSD and were seropositive for AQP4-IgG as determined by cell-based assays (1, 8). Importantly, none of the patients had received any immunotherapy at enrollment. HCs were matched for age, gender, body mass index (BMI), dietary habits, and geographical location. The detailed inclusion and exclusion criteria are presented in **Figure 1**. We excluded subjects with acute infections, gastrointestinal disorders, malignancy, other autoimmune diseases, or those who had taken probiotics, antibiotics, or corticosteroids within one month prior to enrollment.

Immunosuppressant in our study was mycophenolate mofetil (MMF) (20-30mg/kg/day). Demographic information and clinical characteristics, including age, gender, BMI, and other autoantibodies were collected at enrollment. The Medical Ethics Committee of West China Hospital, Sichuan University approved the study, and all participants provided informed consent prior to their inclusion in this study.

### Fecal sample collection and 16S rRNA gene sequencing

All collected fecal samples were immediately cryopreserved at -80°C until processing. Following thawing, genomic DNA was extracted and amplified by PCR using primers (338F: 5’-ACTCCTACGGGAGGCAGCAG-3’; 806R: 5’-GGACTA CHVGGGTWTCTAAT-3’) targeting the V3-V4 hypervariable regions of the bacterial 16S rRNA gene, as previously described (9). The PCR amplicons were purified using an AxyPrep DNA Gel Extraction Kit (Axygen, USA) and prepared for sequencing with the Illumina TruSeq DNA PCR-Free Library Preparation Kit to construct 16S rRNA gene libraries.

### Data processing and bioinformatics analysis

Sequencing was performed on Illumina MiSeq platforms, and the resulting data were analyzed using Quantitative Insights Into Microbial Ecology (QIIME, v2.0) with default parameters for Illumina processing (10). Raw sequences underwent quality filtering prior to assembly, removing: (1) reads with primer mismatches, (2) reads overlaps containing > 5% mismatches, and (3) reads shorter than 100 bp. Qualified paired-end reads were merged using FLASH (v1.2.11) (11). followed by chimera removal with UCHIME. High-quality sequences were clustered into operational taxonomic units (OTUs) at a ≥ 97% similarity threshold using VSEARCH (v2.15.0) (12), with representative sequences selected from each cluster.

Microbial α-diversity was evaluated using Observed species and Chao1 indices (richness), and Shannon and Simpson indices (diversity) (13). β-diversity was evaluated to examine microbial community heterogeneity between NMOSD patients and HCs through principal coordinates analysis (PCoA) based on Bray-Curtis distances and permutational multivariate analysis of variance (PERMANOVA; adonis2 function, 999 permutations).

Linear discriminant analysis effect size (LEfSe) was performed to identify differentially abundant taxa, applying a non-parametric Kruskal-Wallis test (α=0.05) followed by Linear discriminant analysis (LDA) with an effect size threshold of 4.0 (14). Phylogenetic Investigation of Communities by Reconstruction of Unobserved States (PICRUSt2) was employed to predict metagenomic functional content, with Kyoto Encyclopedia of Genes and Genomes (KEGG) pathway analysis performed to identify metabolic pathways associated with taxonomic composition differences (15).

### Sample collection and metabolomics profiling analysis

Fresh fecal samples (around 400 mg) were collected upon enrollment, suspended in preservation buffer (Longseegen Stool Storage Kit, Longsee Biomedical Corp., China) following manufacturer’s protocols, and cryopreserved at -80°C. After homogenization, 200 µL of fecal sample was vacuum-dried and resuspended in 800 µL methanol. All samples were vortexed for 30 s, sonicated for 10 min, and incubated at −20 °C for 2 h to precipitate proteins. After centrifugation (13,000 rpm, 4°C, 15 min), the supernatants were collected, vacuum-dried, and reconstituted in 200 µL methanol/water (1:1, v/v).

Metabolomics analysis was performed by ultra-high-performance liquid chromatography and mass spectrometry (UHPLC-MS). The datasets were analyzed through pattern recognition methods using MetaboAnalyst 3.0. Univariate analysis (t-test) identified statistically significant features between groups, while multivariate partial-least-squares discrimination analysis (PLS-DA) assessed inter-subject variability and highlighted key discriminatory metabolites. Metabolites with PLS-DA-derived VIP > 1.0 were prioritized as key contributors. Pathway analysis of these differential metabolites was performed using the KEGG database (16).

### Statistical analysis

Quantitative data are described as the median (range) or mean ± SD (standard deviation). The Mann-Whitney U test or Student’s t-test were used to compare variables between groups. The false discovery rate (FDR) was calculated to correct for multiple comparisons and assess screened difference variables (17). All analyses were performed using GraphPad Prism (v8.0) (GraphPad Software, Inc., San Diego, CA, USA) or SPSS Statistics software (v27.0) (SPSS Inc., Chicago, IL, USA). A p-value < 0.05 was considered statistically significant.

## Results

Our analysis included twenty-one treatment-naïve NMOSD patients (NT group) and twenty-one matched healthy controls (HCs). Fecal samples were collected from all participants at enrollment. Of these 21 NMOSD patients, 5 subsequently underwent re-examination of fecal samples six months after immunosuppressant treatment (IST group). **Table 1** summarizes the detailed demographic information and clinical characteristics of the participants. The clinical manifestations and severity of each NMOSD patient are shown in **Supplementary Table S1**.

**Table 1:** Demographic and clinical characteristics of patients with NMOSD and healthy controls.

Characteristics	NMOSD	HCs	P values
Stool samples, n	21	21	
Female, n (%)	19 (90.5)	19 (90.5)	1.00
Age, years, mean ± SD	45.1 ± 13.7	47.1 ± 14.2	0.27
BMI, kg/m2, mean ± SD	22.5 ± 3.6	22.97 ± 2.57	0.57
Age at onset, years, mean ± SD	37.3 ± 13.8	–	–
Disease durations, years, mean ± SD	8.5 ± 6.7	–	–
Serum AQP4-IgG, n (%)	21 (100)	–	–
EDSS scores, median, range	3.0 (0-7.0)	–	–

AQP4, aquaporin 4; BMI, body mass index; EDSS, expanded disability status scale; HCs, healthy controls; NMOSD, neuromyelitis optica spectrum disorder; SD, standard deviation.

### Alpha diversity

**Figure 2:**
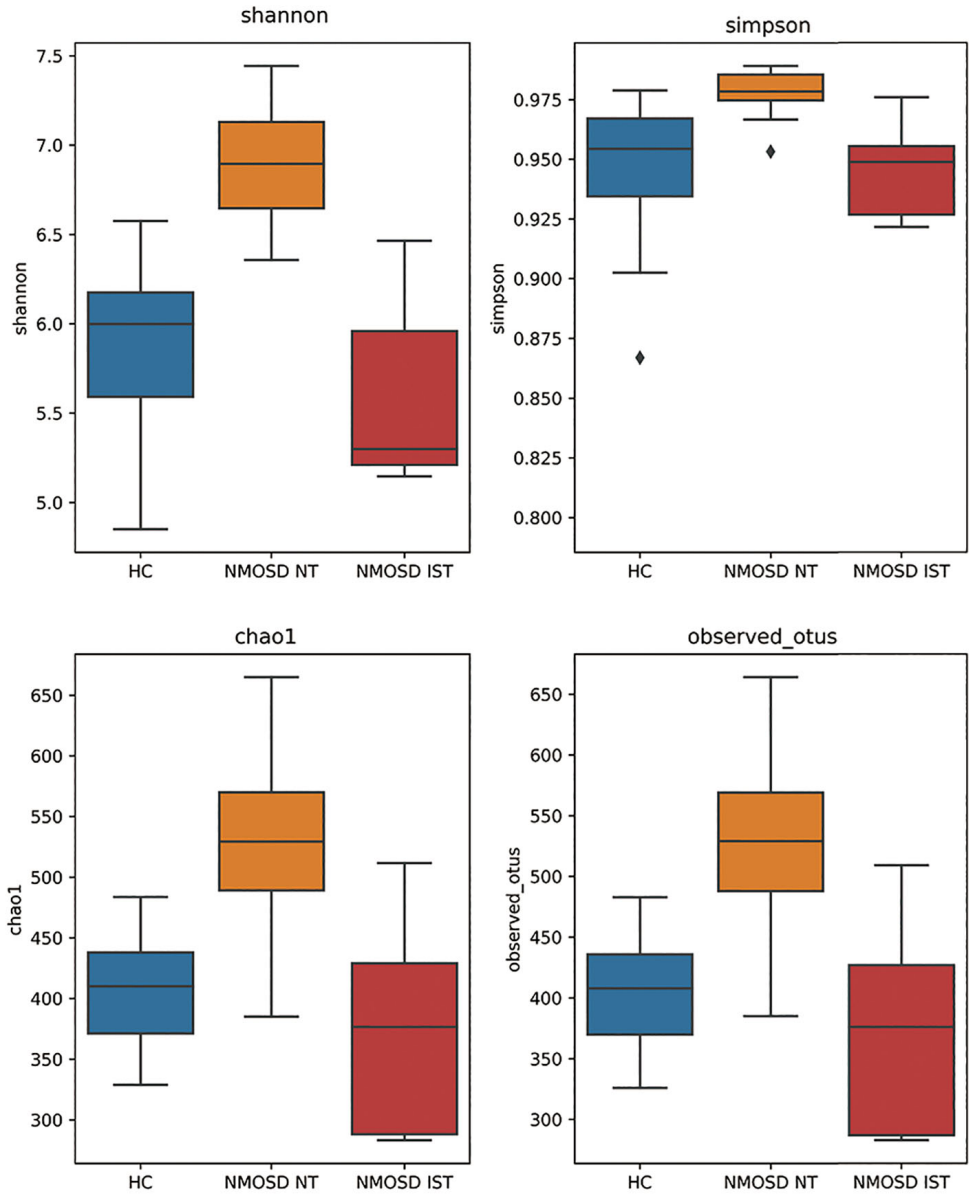
Alpha diversity of gut microbiota in NMOSD patients and HCs. Treatment-naïve NMOSD patients demonstrated significantly higher gut microbial richness and diversity compared to HCs. Following IST, microbial diversity was significantly reduced in NMOSD patients relative to both pretreatment levels and HCs. HC, healthy control; IST, immunosuppressive therapy; NMOSD, neuromyelitis optica spectrum disorder; NT, no treatment.

The α-diversity was compared to assess differences in microbial community structure between treatment-naïve NMOSD patients and HCs. All α-diversity metrics (Chao1, observed OTUs, Shannon index, and Simpson index) were significantly higher in NMOSD patients than in HCs (P < 0.001), indicating greater gut microbial richness and diversity in treatment-naïve NMOSD patients. Furthermore, MMF-treated NMOSD patients showed reduced α-diversity compared to both treatment-naïve patients and HCs, as demonstrated by the Chao1, observed species, Shannon index, and Simpson index (**Figure 2**).

### Beta diversity

**Figure 3:**
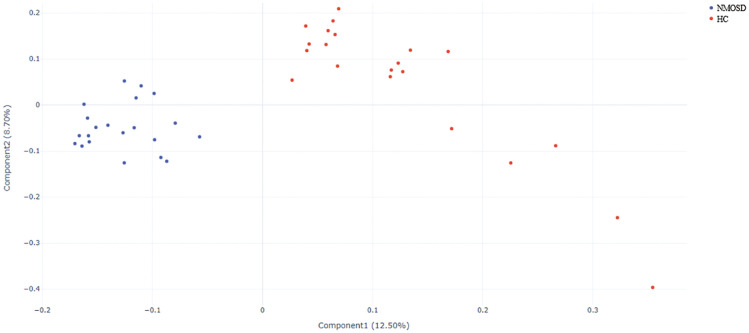
β-Diversity analysis of gut microbiota between treatment-naïve NMOSD patients and HCs. Principal coordinate analysis (PCoA) based on unweighted UniFrac distances revealed distinct clustering patterns between NMOSD patients and HCs. Each point represents an individual sample (red circles = HCs; blue circles = NMOSD), with inter-point distances reflecting microbial community dissimilarity. HC, healthy control; NMOSD, neuromyelitis optica spectrum disorder.

**Figure 4:**
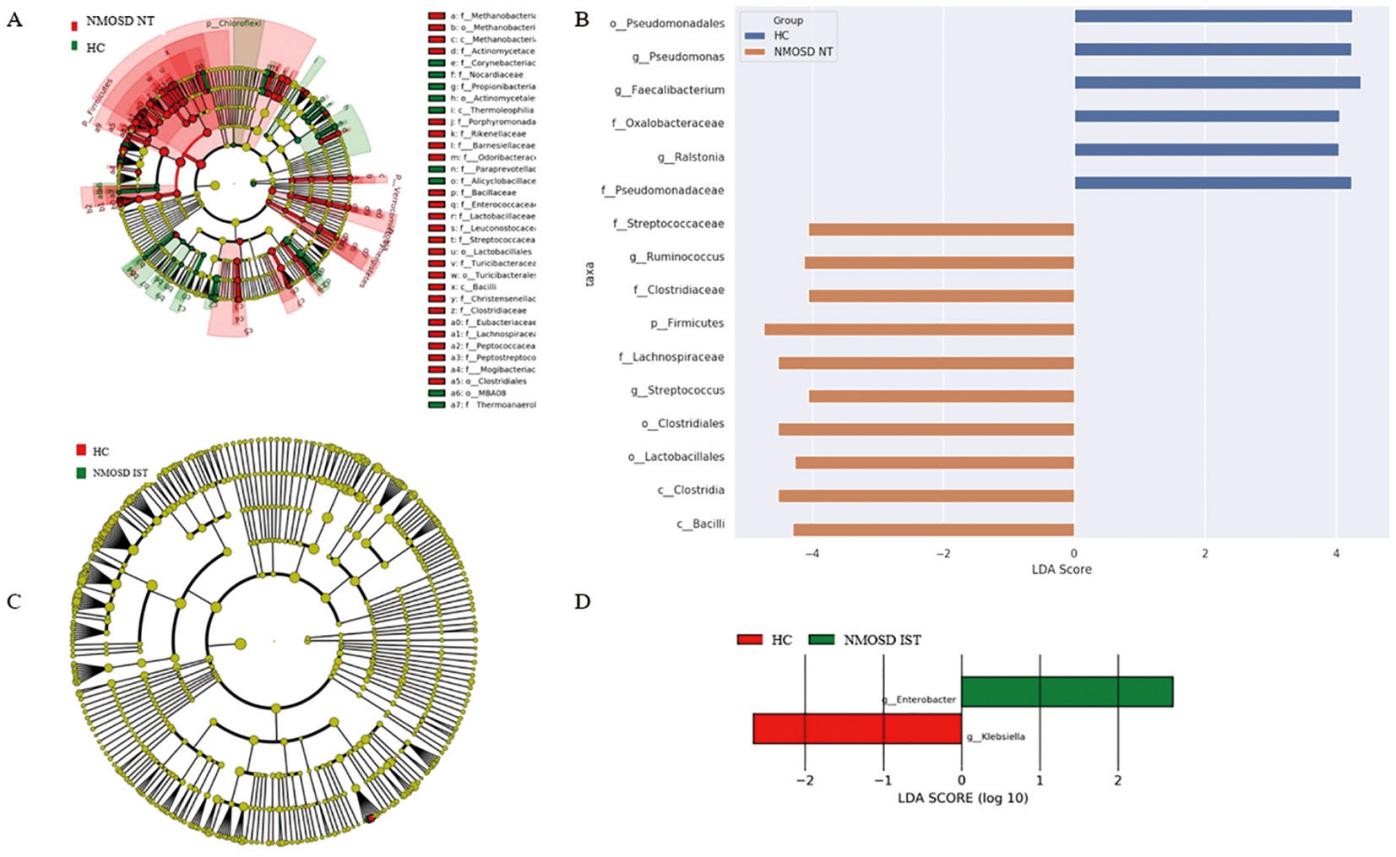
Identification of differentially abundant gut microbes using LEfSe analysis. **(A)** Cladogram showing phylogenetic distribution of gut microbiota in treatment-naïve NMOSD patients versus HCs. **(B)** LDA scores (log10 > 4, p < 0.05) identifying significant bacterial differences between treatment-naïve NMOSD patients and HCs. **(C)** Cladogram showing phylogenetic distribution of gut microbiota in immunosuppressant-treated NMOSD patients versus HCs. **(D)** LDA scores (log10 > 2, p < 0.05) identifying significant bacterial differences between immunosuppressant-treated NMOSD patients and HCs. HC, healthy control; IST, immunosuppressive therapy; LDA, linear discriminant analysis; LEFSe, linear discriminant analysis effect size; NMOSD, neuromyelitis optica spectrum disorder; NT, no treatment.

To compare fecal microbial community structures between groups, β-diversity was assessed by calculating pairwise Bray-Curtis distances. PCoA based on the resulting distance matrices revealed distinct clustering between treatment-naïve NMOSD patients and HCs, with clear separation along the primary axes (**Figure 3**). PERMANOVA confirmed significant compositional differences between groups (P < 0.001, R² = 0.13). LDA further identified phylogenetic clustering patterns. Specific genera (*Streptococcus* and *Ruminococcus*) showed significantly higher abundance in NMOSD patients compared to HCs, while *Faecalibacterium*, *Pseudomonas*, and *Ralstonia* were more abundant in HCs (LDA score > 4, P < 0.05; **Figure 4A**). Collectively, intestinal microbiota profiling demonstrated gut dysbiosis in NMOSD patients, characterized by increased α-diversity and altered community composition. These results establish significant differences between the gut microbiota of NMOSD patients and HCs.

A comparative analysis of IST groups revealed only two significant differences in taxonomic biomarkers at the genus level between immunosuppressant-treated NMOSD patients and HCs. Specifically, *Enterobacter* was enriched in the NMOSD IST group (LDA score = 2.70, P < 0.05), whereas *Klebsiella* showed high abundance in HCs (LDA score = 2.66, P < 0.05) (**Figure 4B**).

### Community analysis

**Figure 5:**
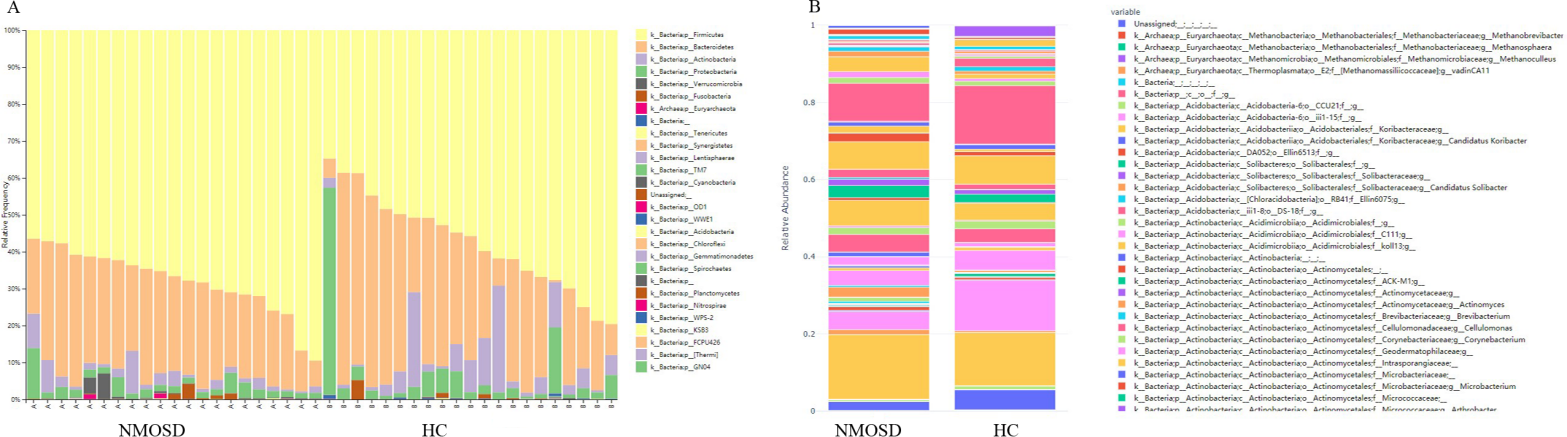
Gut microbiota community structure comparison between NMOSD patients and HCs. **(A)** Phylum-level and **(B)** genus-level composition of gut microbial communities in treatment-naïve NMOSD patients versus HCs. HC, healthy control; NMOSD, neuromyelitis optica spectrum disorder.

Community-level analysis of gut microbiota revealed that Bacteroidetes, Firmicutes, Actinobacteria, and Proteobacteria were the predominant phyla in both treatment-naïve NMOSD patients and healthy controls (HCs), collectively comprising > 90% of the intestinal microbiota (**Figure 5A**). At the genus level, *Bacteroides*, *Faecherlibacterium*, *Prevotella*, *Roseburia*, and *Blautia* represented the five most abundant taxa (**Figure 5B**).

### Overall metabolomics analysis

**Figure 6:**
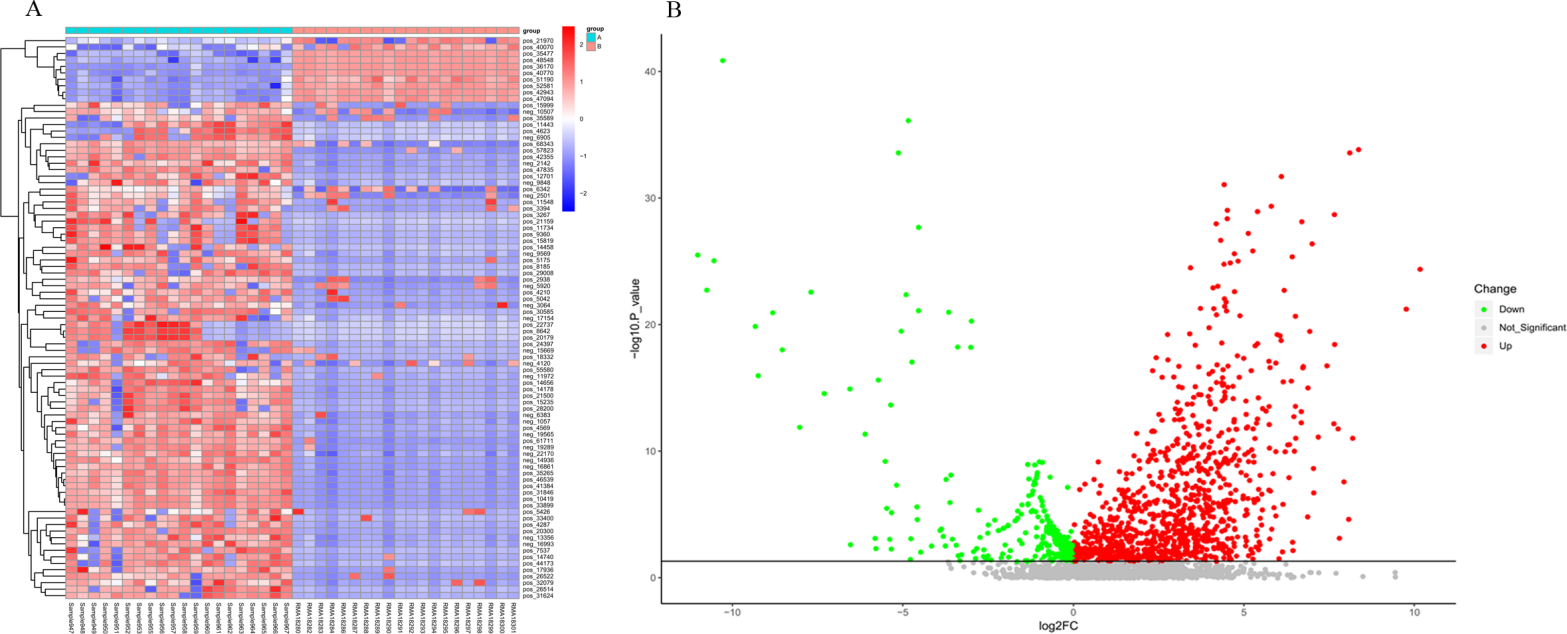
Metabolic profiles in treatment-naïve NMOSD patients versus HCs. **(A)** Heatmap and **(B)** volcano plot displaying fecal metabolite profiles. Rows represent individual metabolites, columns represent study participants. Color gradients indicate relative metabolite abundance (red: increased; blue: decreased). HC, healthy control; NMOSD, neuromyelitis optica spectrum disorder.

**Figure 7:**
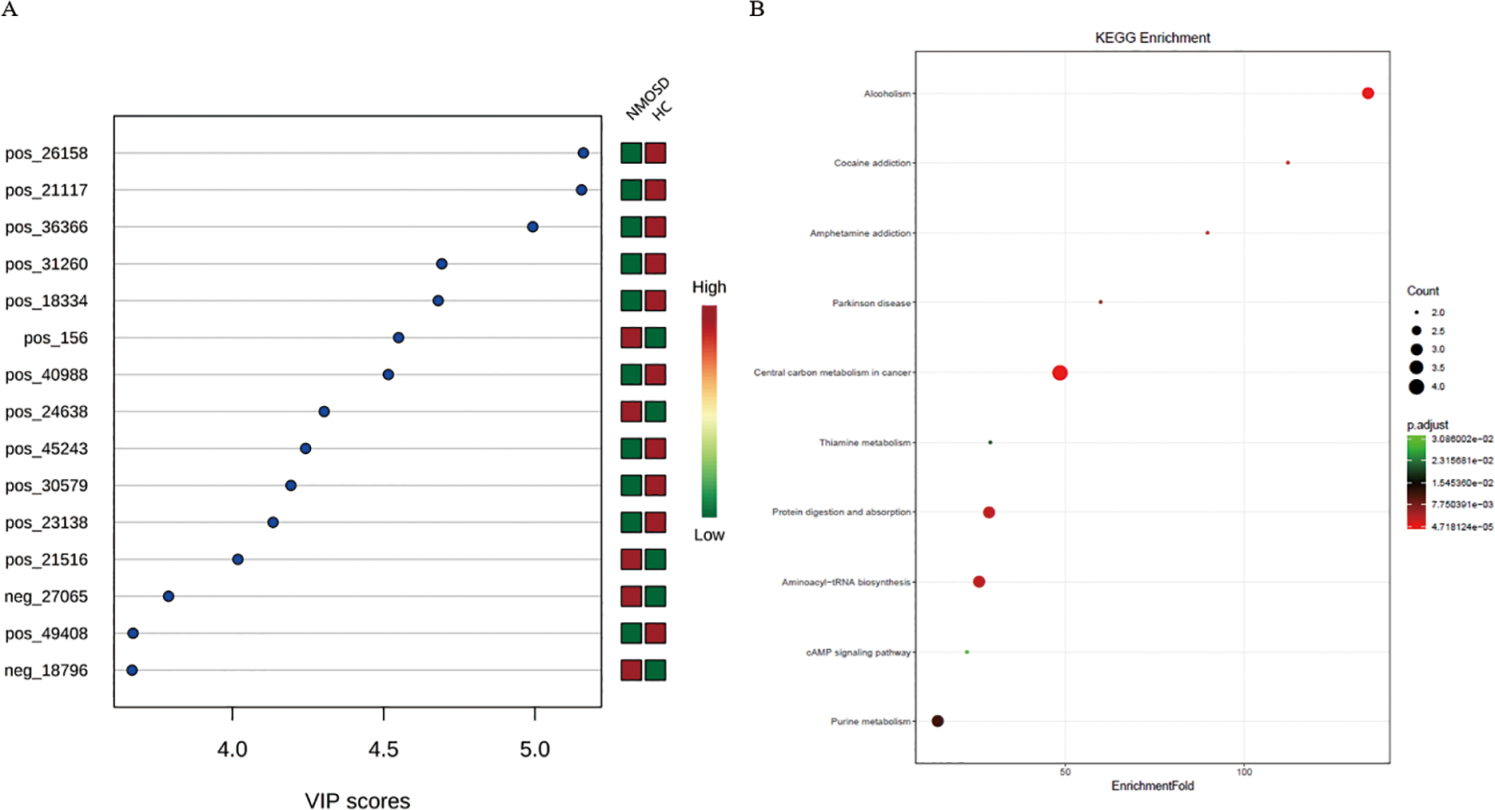
PLS-DA VIP analysis of differential metabolites in treatment-naïve NMOSD patients versus HCs. **(A)** VIP scores of significant metabolites (red: increased; green: decreased levels). **(B)** Enriched metabolic pathways showing pathway impact versus -log10(p-value) (top 10 pathways highlighted). HC, healthy control; NMOSD, neuromyelitis optica spectrum disorder; PLS-DA, partial least squares discriminant analysis; VIP, variable importance in projection.

The metabolomes of 21 fecal samples from NMOSD patients and HCs were characterized and compared. Features with VIP scores > 1.0 in multivariate analysis and p value < 0.05 in univariate analysis were identified as the most important metabolites and were visualized in a heatmap (**Figure 6A**) and volcano plot (**Figure 6B**). A total of 19 key metabolites involved in amino acid, lipid, purine, and vitamin metabolism were altered in the feces of NMOSD patients compared with HCs (**Table 2**). The levels of D-Proline, L-Tyrosine, L-Methionine, L-Glutamate, N&omega-Acetylhistamine, Val-Ala, L-Leucyl-L-Alanine, Val-Leu, Thr-Ile, Leu-Ile, Phe-Ile, Adenosine, Adenine, Hypoxanthine, Linoelaidic Acid, Palmitoyl Ethanolamide, 5(S)-HETE, and vitamin B1 were elevated in NMOSD fecal samples. In contrast, DL-phenylalanine levels were decreased in NMOSD (**Figure 7A**).

**Table 2:** Fecal identified differential metabolites between NMOSD patients and healthy controls.

Metabolite	P value	VIP	FC	Pathways
D-Proline	<0.001	1.615	0.027	Amino acid metabolism
L-Tyrosine	<0.001	1.734	5.068	Amino acid metabolism
L-Methionine	<0.001	1.775	3.099	Amino acid metabolism
L-Glutamate	<0.001	1.459	0.985	Amino acid metabolism
DL-Phenylalanine	0.019	1.351	-0.487	Amino acid metabolism
Val Ala	<0.001	1.425	3.998	Amino acid metabolism
L-Leucyl-L-Alanine	<0.001	1.815	5.301	Amino acid metabolism
Val Leu	<0.001	1.668	0.462	Amino acid metabolism
Thr Ile	<0.001	1.377	4.322	Amino acid metabolism
Leu Ile	<0.001	1.577	4.455	Amino acid metabolism
Phe Ile	<0.001	1.639	4.598	Amino acid metabolism
Adenosine	<0.001	1.694	3.634	Purine metabolism
Adenine	<0.001	1.378	1.714	Purine metabolism
Hypoxanthine	<0.001	1.440	3.691	Purine metabolism
Linoelaidic Acid	<0.001	1.651	1.260	Fatty acid metabolism
Palmitoyl Ethanolamide	<0.001	2.124	3.666	Fatty acid metabolism
5(S)-HETE	0.004	1.468	2.322	Fatty acid metabolism
N&omega-Acetylhistamine	0.017	1.005	0.819	Other

FC, fold change; NMOSD, neuromyelitis optica spectrum disorder**;** VIP, variable importance in the projection.

### Metabolic pathway analysis

To identify biologically meaningful patterns based on the metabolomics data in feces, pathway profiling was conducted through the KEGG metabolic library using Metaboanalyst 3.0. Taken together, 44 gut microbe-related KEGG pathways were identified as differentially enriched between NMOSD group and HC group, among which 10 remained significant after FDR correction (FDR-adjusted P < 0.05, **Figure 7B**). Elevated L-glutamate and L-tyrosine were enriched in alcoholism, central carbon metabolism in cancer, protein digestion and absorption, cocaine addiction, aminoacyl-tRNA biosynthesis, and amphetamine addiction. Meanwhile, L-tyrosine was also associated with Parkinson’s disease and thiamine metabolism. Increased L-methionine was involved in central carbon metabolism in cancer, protein digestion and absorption, and aminoacyl-tRNA biosynthesis. Elevated adenosine was linked to alcoholism, Parkinson’s disease, purine metabolism, and the cAMP signaling pathway. Higher hypoxanthine and thiamine levels were associated with purine metabolism and thiamine metabolism, respectively. Additionally, elevated (S)-lactate was enriched in central carbon metabolism in cancer and the cAMP signaling pathway, while increased adenine was linked to purine metabolism.

### The impact of immunosuppressants on the gut microbiota of NMOSD patients

**Figure 8:**
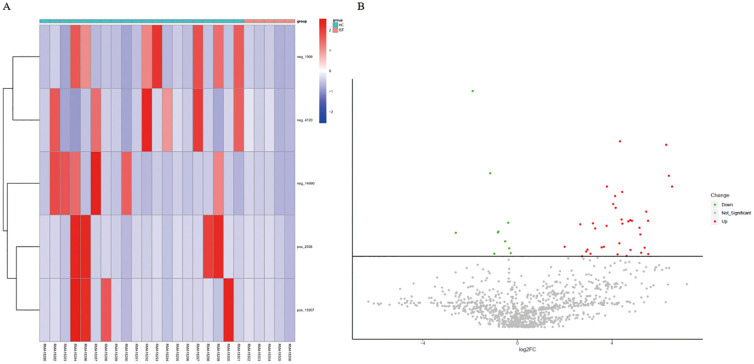
Metabolite profiles in immunosuppressant-treated NMOSD patients versus HCs. **(A)** Heatmap and **(B)** volcano plot displaying fecal metabolite profiles. Rows correspond to individual metabolites; columns represent study participants. Color gradients indicate relative abundance (red: increased; blue: decreased). HC, healthy control; IST, immunosuppressive therapy; NMOSD, neuromyelitis optica spectrum disorder.

**Figure 9:**
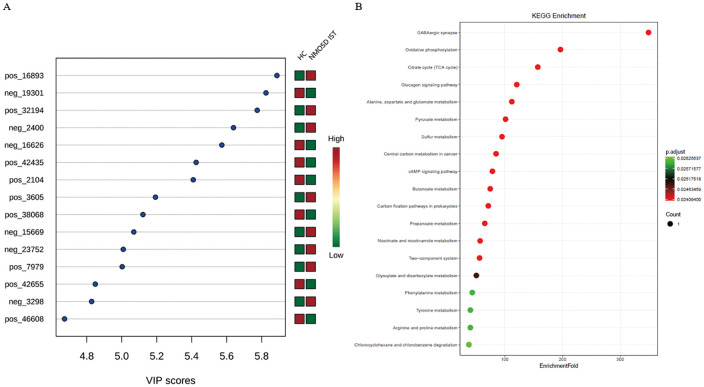
PLS-DA VIP analysis of differential metabolites in immunosuppressant-treated NMOSD patients versus HCs. **(A)** VIP scores of significant metabolites (red: increased; green: decreased levels). **(B)** Enriched metabolic pathways showing pathway impact versus -log10(p-value) (top 2 pathways highlighted). HC, healthy control; NMOSD, neuromyelitis optica spectrum disorder; PLS-DA, partial least squares discriminant analysis; VIP, variable importance in projection.

After six months of immunosuppressive therapy, fecal samples from treatment-naïve NMOSD patients were re-collected and analyzed. Metabolomics analysis revealed that only two key metabolites were altered in the feces of NMOSD patients receiving immunosuppressants (IST group) compared with HCs (**Table 3**), as shown in a heatmap (**Figure 8A**) and volcano plot (**Figure 8B**). The levels of D-proline and oleic acid ethyl ester were elevated in the IST group (**Figure 9A**). In total, 19 gut microbe-related KEGG pathways were differentially enriched between the IST and HC groups after FDR correction (P < 0.05; **Figure 9B**), including tyrosine metabolism, citrate cycle (TCA cycle), oxidative phosphorylation, alanine/aspartate/glutamate metabolism, pyruvate metabolism, butanoate metabolism, carbon fixation in prokaryotes, sulfur metabolism, cAMP signaling pathway, GABAergic synapse, glucagon signaling pathway, central carbon metabolism in cancer, propanoate metabolism, nicotinate/nicotinamide metabolism, two-component system, glyoxylate/dicarboxylate metabolism, phenylalanine metabolism, arginine/proline metabolism, and chlorocyclohexane/chlorobenzene degradation. D-proline was enriched in arginine/proline metabolism, while the remaining pathways were associated with increased succinate levels.

**Table 3:** Fecal identified differential metabolites between NMOSD IST group patients and healthy controls.

Metabolite	P value	VIP	FC	Pathways
D-Proline	0.043	2.360	5.233	Amino acid metabolism
Oleic Acid ethyl ester	0.049	2.085	4.619	Fatty acid metabolism

FC, fold change; IST, immunosuppressive therapy, NMOSD, neuromyelitis optica spectrum disorder; VIP, variable importance in the projection.

## Discussion

In the present study, we identified a striking gut microbiota imbalance in treatment-naïve NMOSD patients compared to HCs, along with dysregulated microbiota-associated metabolic pathways. Community analysis and β-diversity revealed significant differences in microbial composition between the two groups. The α-diversity of fecal microbiota in NMOSD patients was significantly higher than that in HCs, suggesting a potential link between gut microbiota and NMOSD pathogenesis. Our findings align with prior Chinese cohort studies (18, 19) reporting altered fecal microbiome α-diversity in NMOSD patients versus HCs. Although higher microbiota diversity is generally beneficial, elevated diversity and richness have also been observed in diseases like depression (20) and autism (21).

In our study, an overgrowth of opportunistic pathogens (e.g., *Streptococcus* and *Ruminococcus*) characterizes gut dysbiosis in NMOSD patients. Prior studies in Chinese NMOSD cohorts also reported associations with *Streptococcus* (7, 18, 19). Notably, Gong et al. found *Streptococcus* abundance positively correlated with disease severity (18). The immunomodulatory mechanism of *Streptococcus* remains unclear; however, its overrepresentation correlates with reduced short-chain fatty acid (SCFA) levels in NMOSD patients, which may enhance CD4+ T cell activity and pro-inflammatory responses (18, 22). In addition, increased *Streptococcus* abundance may promote inflammation by elevating pro-inflammatory cytokines (TNF-α, IL-6, and IFN-γ), which can also induce Th1/Th17 cell differentiation in humans (20, 23). Furthermore, *Streptococcus* species such as *S. pyogenes* were shown to reduce Treg frequency in tumor microenvironments and impair Treg function via APC-derived IL-12 (24). These findings suggest *Streptococcus* may play a pivotal role in NMOSD pathogenesis. This contrasts with prior reports implicating *Clostridium perfringens* through molecular mimicry (25). We propose that genetic and environmental differences between Caucasian and Asian populations may underlie these discrepant findings.

We observed increased *Ruminococcus* abundance in the gut microbiota of NMOSD patients, though its mechanistic role requires further investigation. Xie et al. similarly reported elevated *Ruminococcaceae* levels during NMOSD acute phases (19). *Ruminococcus* is an anaerobic bacterial genus with context-dependent roles in disease. While some species promote health through SCFA production, others may exacerbate disease when overabundant (26). This genus is enriched in multiple disorders, including Crohn’s disease, inflammatory bowel disease (IBD), spondylarthritis, and asthma (27). Notably, IBD patients exhibit transient *Ruminococcus* blooms correlating with disease activity (28), and its abundance associates with pro-inflammatory cytokines (e.g., IL-6, TNF-α) and LPS levels (29). Together with prior studies, our findings suggest that *Streptococcus* and *Ruminococcus* overgrowth may contribute to NMOSD pathogenesis via inflammatory mechanisms.

Furthermore, our data revealed depletion of beneficial commensal microbes (e.g., *Faecalibacterium*) in NMOSD patients. As a dominant bacterial genus in the healthy human gut, *Faecalibacterium* suppresses inflammation by producing SCFAs and IL-10. It also enhances gut barrier integrity, inhibiting pathogen invasion (30). The roles of *Pseudomonas* and *Ralstonia* in NMOSD remain unclear and warrant investigation. These findings suggest that gut microbiota dysbiosis, characterized by diminished commensal bacteria and increased opportunistic pathogens, may contribute to NMOSD pathogenesis through dysregulation of inflammatory mediators. However, further studies are needed to establish whether this dysbiosis initiates NMOSD or arises secondary to the disease.

To date, little is known about the potential link between gut microbiome metabolic pathways and NMOSD pathogenesis. Functional analyses revealed that gut microbiota alterations may contribute to NMOSD development through associated metabolic pathways. In this study, metabolomic profiling of fecal samples identified 19 significantly altered metabolites in treatment-naïve NMOSD patients. Furthermore, PICRUSt analysis uncovered 10 dysregulated microbiome-associated metabolic pathways related to NMOSD. Notably, elevated fecal levels of glucogenic amino acids (including D-proline, L-glutamate, and L-methionine) and glucogenic/ketogenic amino acids (particularly L-tyrosine) were observed in NMOSD patients. These findings suggest potential disruptions in glucose and energy metabolism, as these amino acids may serve as alternative energy substrates. Glutamate metabolism has been previously reported to modulate both resting and activated T-cell function (31). Proline, a key component of mucus glycoproteins in colonic epithelial cells (32), showed elevated levels in stool samples, potentially indicating alterations in mucin synthesis and intestinal barrier function. These findings suggest a potential link between amino acid metabolism and gut homeostasis. We hypothesize that impaired amino acid absorption may lead to their accumulation in the intestinal lumen. Most notably, the significantly enriched aminoacyl-tRNA biosynthesis pathway, involving L-methionine, L-tyrosine and D-proline, indicates dysregulated amino acid turnover and protein biosynthesis in NMOSD patients.

Our analysis revealed skewed vitamin B metabolism in fecal samples from NMOSD patients. Costliow et al. suggested that under conditions of thiamine limitation, the biosynthesis of thiamine is crucial for the growth and competitive fitness of *B. thetaiotaomicron* (33), a *Bacteroides* species (phylum Bacteroidetes) in the human gut microbiota. We hypothesize that elevated fecal thiamine levels in NMOSD patients may sustain microbial survival by altering gut vitamin B metabolism. However, the immunomodulatory role of vitamin B remains poorly understood. Investigating vitamin B-mediated immune regulation could provide insights into NMOSD pathogenesis and reveal novel therapeutic targets.

Metabolites associated with purine metabolism were dysregulated in NMOSD patients compared to HCs, including hypoxanthine, adenine, and adenosine. Adenosine is a key immunosuppressive mediator secreted by Treg cells, which suppresses self-reactive immune responses, promotes transplant tolerance, and mitigates autoimmune disorders (34–36). Additionally, lipid metabolism, which regulates critical cellular processes such as proliferation, differentiation, inflammation, and apoptosis, was significantly altered in NMOSD patients. Key perturbed metabolites in this pathway included linoelaidic acid, palmitoyl ethanolamide, and 5(S)-HETE. These findings suggest a potential link between lipid carbon-chain metabolism and NMOSD pathology. Thus, our study provides mechanistic insights into how gut microbiota-derived metabolic changes may contribute to NMOSD pathogenesis.

Notably, metabolism can be influenced by drug intake (37). Intriguingly, in the present study, not only did the gut microbiome composition in treatment-naïve NMOSD patients closely resemble that of HCs after immunosuppressive therapy, but principal component analysis also revealed significant differences in overall metabolic profiles between NMOSD patients and HCs. Following therapy, the gut microbial metabolites in these patients became nearly identical to those in HCs. This suggests that metabolic alterations may arise not only from the disease itself but also from therapeutic interventions. A limitation of this study is the small cohort of treatment-naïve NMOSD patients, which limits the statistical power to precisely evaluate medication-induced changes in fecal metabolites. Future studies should enroll a larger treatment-naïve cohort to better delineate treatment-specific metabolic shifts.

Nevertheless, our study has several limitations. First, as a single-center case-control study, the sample size was limited, particularly in the longitudinal immunosuppressive treatment group. This precluded robust subgroup analyses to assess how disease status or clinical presentation specifically affects microbiota abundance. Second, although early longitudinal data from five patients suggested clinical benefits from immunosuppressants, the scarcity of serial samples prevented a comparative analysis of microbial or metabolomic dynamics between treatment responders and non-responders. Third, while cross-sectional analyses indicate that therapy is associated with a microbiota shift towards a state resembling healthy controls, the most direct evidence would come from paired pre- and post-treatment samples. The limited availability of such longitudinal specimens restricted our ability to perform this definitive within-patient analysis. Therefore, our findings necessitate further validation in larger, multi-center prospective studies with comprehensive longitudinal sampling. In addition, dietary habits may influence microbial community dynamics; thus, standardized dietary questionnaires need to be incorporated into gut microbiota research. Finally, the follow-up period of this longitudinal cohort study was limited to 6 months, which may not fully capture the long-term effects of immunosuppressive therapy on gut microbiota in NMOSD patients.

## Conclusions

In conclusion, our research reveals that intestinal dysbiosis and aberrant metabolic pathways exist in NMOSD patients. Immunotherapy may shift the gut microbiota of NMOSD patients toward that of HCs. These findings could not only help elucidate the underlying mechanism by which microbiome dysbiosis contributes to NMOSD pathogenesis but also provide insights into potential individualized treatments for the disease. Further studies with larger sample sizes and extended follow-up durations are warranted to confirm our findings.

## Data Availability

The raw data supporting the conclusions of this article will be made available by the authors, without undue reservation.

## Supplementary material

Supplementary Table 1 Clinical manifestations and severity of each patient with NMOSD. AQP4, aquaporin 4; APS, Area postrema syndrome; BS, brainstem syndrome; EDSS, expanded disability status scale; NMOSD, neuromyelitis optica spectrum disorder; ON, optic neuritis; TM, transverse myelitis.
