# Supplementary material for: Dysbiosis of intestinal microbiota in patients with neuromyelitis optica spectrum disorders
Source: Front Immunol. 2026 Feb 27;17:1747643. doi: 10.3389/fimmu.2026.1747643 (PMC12982100; doi:10.3389/fimmu.2026.1747643)
Supplement: Supplementary Table 1 — Clinical manifestations and severity of each patient with NMOSD. AQP4, aquaporin 4; APS, Area postrema syndrome; BS, brainstem syndrome; EDSS, expanded disability status scale; NMOSD, neuromyelitis optica spectrum disorder; ON, optic neuritis; TM, transverse myelitis. [file Table1.docx]

**Table S1** Clinical manifestations and severity of each patient with NMOSD

| Patiens | Disease status | Disease durations at sampling, years | EDSS at sampling | Phenotype | Serum AQP4-IgG at sampling | Medications at sampling | Sampling timepoint |
| --- | --- | --- | --- | --- | --- | --- | --- |
| No.1 | Onset | 0.44 | 4.5 | ON+TM | 1:32 | None | Remission |
| No.2 | Relapse | 9.57 | 4 | ON+TM | 1:32 | None | Remission |
| No.3 | Relapse | 5.30 | 5.5 | ON+TM | 1:3200 | None | Remission |
| No.4 | Relapse | 0.27 | 4 | ON | 1:100 | None, prior to steroid pulse | Acute relapse |
| No.5 | Onset | 0.02 | 6 | TM | 1:320 | None, prior to steroid pulse | Acute attack |
| No.6 | Relapse | 2.85 | 3 | ON | 1:32 | None, prior to steroid pulse | Acute relapse |
| No.7 | Relapse | 15.85 | 3 | ON+TM | 1:32 | None | Remission |
| No.8 | Relapse | 15.13 | 3 | ON+TM | 1:100 | None, prior to steroid pulse | Acute relapse |
| No.9 | Relapse | 0.59 | 3 | ON+TM | 1:100 | None | Remission |
| No.10 | Relapse | 20.52 | 4.5 | ON+TM | 1:10 | None | Remission |
| No.11 | Relapse | 0.35 | 6.5 | BS+TM | 1:32 | None, prior to steroid pulse | Acute relapse |
| No.12 | Onset | 0.06 | 2 | TM | 1:1000 | None, prior to steroid pulse | Acute attack |
| No.13 | Relapse | 3.25 | 0 | ON+TM | 1:32 | None | Remission |
| No.14 | Relapse | 15.88 | 6 | ON+TM | 1:32 | None, prior to steroid pulse | Acute relapse |
| No.15 | Relapse | 14.86 | 3 | ON+TM | 1:320 | None | Remission |
| No.16 | Relapse | 11.0 | 0 | TM | 1:100 | None | Remission |
| No.17 | Relapse | 9.78 | 0 | ON+TM | 1:10 | None | Remission |
| No.18 | Relapse | 5.93 | 3 | APS+ON+TM | 1:100 | None | Remission |
| No.19 | Relapse | 18.42 | 3 | ON+TM | 1:32 | None | Remission |
| No.20 | Onset | 4.35 | 0 | TM | 1:10 | None | Remission |
| No.21 | Onset | 5.52 | 1 | TM | 1:32 | None | Remission |

AQP4, aquaporin 4; APS, Area postrema syndrome; BS, brainstem syndrome; EDSS, expanded disability status scale; NMOSD, neuromyelitis optica spectrum disorder; ON, optic neuritis; TM, transverse myelitis.
